# Impact of mental disorders on the risk of atrial fibrillation in patients with diabetes mellitus: a nationwide population-based study

**DOI:** 10.1186/s12933-022-01682-7

**Published:** 2022-11-17

**Authors:** Nan Young Bae, So-Ryoung Lee, Eue-Keun Choi, Hyun Jin Ahn, Hyo-Jeong Ahn, Soonil Kwon, Kyung-Do Han, Kyu-Na Lee, Seil Oh, Gregory Y. H. Lip

**Affiliations:** 1 Department of Internal Medicine, Seoul National University Hospital, 101 Daehak-ro, Jongno-gu, Seoul, 03080 Republic of Korea; 2 Department of Internal Medicine, Seoul National University College of Medicine, Seoul, Republic of Korea; 3 Department of Statistics and Actuarial Science, Soongsil University, Seoul, Republic of Korea; 4 Liverpool Centre for Cardiovascular Science, University of Liverpool and Liverpool Chest & Heart Hospital, Liverpool, UK; 5 Department of Clinical Medicine, Aalborg University, Aalborg, Denmark

**Keywords:** Atrial fibrillation, Diabetes mellitus, Mental disorders

## Abstract

**Background:**

It is unclear whether mental disorders are an independent risk factor for atrial fibrillation (AF) in patients with diabetes. We aimed to investigate whether patients with diabetes who have mental disorders have an increased risk for AF.

**Methods:**

Using the Korea National Health Insurance Service database, we enrolled 2,512,690 patients diagnosed with diabetes without AF between 2009 and 2012. We assessed five mental disorders: depression, insomnia, anxiety, bipolar disorder, and schizophrenia. Newly diagnosed AF was identified during the follow-up period, and multivariate Cox regression analysis was performed.

**Results:**

Among the 2,512,690 patients (mean age 57.2 ± 12.3 years; 60.1% men), 828,929 (33.0%) had mental disorders. Among the five mental disorders, anxiety (68.1%) was the most common, followed by insomnia (40.0%). During a median follow-up duration of 7.1 years, new-onset AF was diagnosed in 79,525 patients (4.66 per 1,000 person-years). Patients with diabetes who had mental disorders showed a higher risk for AF (adjusted hazard ratio [HR] 1.19; 95% confidence interval [CI] 1.17–1.21; p-value < 0.001). Depression, insomnia, and anxiety were significantly associated with higher risk for AF (adjusted HR [95% CI]: 1.15 [1.12–1.17], 1.15 [1.13–1.18], and 1.19 [1.67–1.21], respectively; all p-values < 0.001), whereas bipolar disorder and schizophrenia were not.

**Conclusions:**

Mental disorders, especially depression, insomnia, and anxiety, were associated with an increased risk for AF in patients with diabetes. Greater awareness with a prompt diagnosis of AF should be considered for patients with both DM and mental disorders.

**Supplementary Information:**

The online version contains supplementary material available at 10.1186/s12933-022-01682-7.

## Background

Not only various medical conditions but also mental disorders increase the risk of cardiovascular diseases (CVDs) [1–4]. Among various CVDs, atrial fibrillation (AF) is the most common cardiac arrhythmia in adults. The prevalence and incidence of AF are continuously rising with population aging [5]. AF is one of the major CVDs because it lowers patients’ quality of life and increases the risk of ischemic stroke, heart failure hospitalization, cognitive impairment, and all-cause death [5]. The risk of AF is significantly higher in patients with mental disorders according to previous observational studies [6, 7] and mental disorders are associated with increased risks of stroke and bleeding in AF [8, 9]. Diabetes mellitus (DM) is a type of CVDs that has a much higher prevalence than AF and also significantly increases the risk of macrovascular, microvascular complications, and all-cause death [10]. DM is also well-known as a risk factor for AF and a risk factor for increasing the risk of stroke and AF-related complications in patients with AF [11]. Poorly controlled DM was associated with a higher risk of ischemic stroke in patients with AF [11, 12]. Therefore, recognizing and managing risk factors for AF in diabetic patients would be an important part of the management of diabetic patients.

DM is a disease that requires chronic management, and it is known that patients have lower life quality, and the prevalence of mental disorders such as depression and insomnia is higher than that of the general population [13, 14]. However, comprehensive analysis on whether accompanying mental disorders in patients with DM increases their risk for new-onset AF is limited. In this study, we aimed to evaluate the impact of mental disorders, including depression, insomnia, anxiety, bipolar disorder, and schizophrenia, on the risk of new-onset AF in patients with DM using a nationwide population-based cohort.

## Methods

We performed a nationwide population-based cohort study using data from the National Health Information Database (NHID) in the Republic of Korea (hereafter denoted as Korea). The Korean NHID was created by the Korean National Health Insurance System (NHIS), which is the sole medical insurance system managed by the Korean government and covers the entire Korean population. All insured adults in the Korean NHIS are required to undergo regular health examinations. Health screening information from the Korean NHID contains detailed lifestyle questionnaires, laboratory results, and anthropometric measurements [15]. Diagnoses are also included in the Korean NHID and coded as the Korean Standard Classification of Diseases Version 7 based on the International Classification of Diseases 10^th^ Revision (ICD-10). Using the Korean NHID was possible because our study protocols were approved by both the governed official review committee and the institutional review board at Seoul National University Hospital (E-2205-006-1320).

### Study population

We identified 2,746,079 patients with diabetes who underwent health checkups between January 2009 and December 2012. A total of 117,836 patients aged < 20 years or with missing data from health examinations were excluded. Further, those who had prevalent AF or were diagnosed with AF within 1 year of the index date were excluded (n = 115,553). Finally, 2,512,690 patients with DM were included in this study.

### Five mental disorders

Depression and anxiety are the most common diseases among mental disorders [16], and bipolar disorder and schizophrenia are known to be accompanied by depression and anxiety [17–20]. Although depression or anxiety is known to be related to AF, many studies have shown conflicting results [21–23]. There are few studies about an association between AF and bipolar disorder or schizophrenia. Therefore, we analyzed depression, anxiety, bipolar disorder, and schizophrenia. Additionally, sleep apnea is known as a risk factor for AF while inducing sleep fragmentation [24], and insomnia was also analyzed.

### Covariates

Patients with diabetes were defined as having (1) DM (ICD-10 codes, E11-E14) diagnosed before the index date with anti-diabetic drugs prescribed or (2) fasting glucose level > 126 mg/dL. A previous diagnosis of a mental disorder was defined as the presence of an insurance claim with ICD-10 codes for mental disorders within 5 years before the index date. Mental disorders included depression, bipolar disorder, schizophrenia, insomnia, and anxiety. The detailed operational definitions are summarized in Additional file 1: Table S1. Baseline characteristics of the study participants, including age, sex, body mass index (BMI), fasting glucose, demographics, and comorbidities (such as hypertension, dyslipidemia, chronic kidney disease, congestive heart failure, obstructive sleep apnea, and thyroid disease) were obtained. Data on lifestyle behaviors, including smoking status, alcohol consumption, and physical activity, were collected using a self-reported questionnaire. BMI and fasting glucose levels were obtained from the health screening examination database. Detailed information regarding the definitions of comorbidities and demographic data is summarized in the supplementary file (Additional file 1: Table S1).

### Study outcomes and follow-up

The primary outcome in this study was new-onset AF during the follow-up period. New-onset AF was identified using diagnostic codes (ICD-10 codes I480–I484 and I489) and satisfied with one diagnosis during hospitalization or at least two diagnoses at an outpatient clinic while receiving follow-up care [25, 26]. Participants were followed up from the index date to the date of occurrence of AF, death, or the end of the study period (December 31, 2018), whichever came first.

### Statistical analysis

Patients with diabetes were divided into two groups according to the presence of prevalent mental disorders. The baseline characteristics are reported as mean ± standard deviation for continuous variables and as number and percentage for categorical variables. To compare the two groups for significant differences in baseline characteristics, the t-test was used for continuous variables and the Fisher exact or chi-squared test for categorical variables. The incidence of new-onset AF in patients with diabetes who had mental disorders was compared with that in those without mental disorders. The incidence rates of new-onset AF were calculated as the number of cases divided by 1000 person-years (PY) at risk during the follow-up period. The five types of mental disorders were analyzed separately. Kaplan–Meier curves were generated for time to the occurrence of AF, and the log-rank test was used to compare the cumulative incidence rates of AF in the groups.

To evaluate the association between mental disorders and new-onset AF, hazard ratios (HRs) and 95% confidence intervals (CIs) were calculated using a Cox proportional hazard regression model. Model 1 represented an unadjusted risk, while Model 2 was adjusted for age and sex, and Model 3 was adjusted for age, sex, BMI, low-income level, smoking status, alcohol consumption status, regular physical activity, hypertension, dyslipidemia, chronic kidney disease, heart failure, obstructive sleep apnea, thyroid disease, fasting glucose, DM duration (≥ 5 years), insulin use, and oral hypoglycemic agents (≥ 3 agents). To assess the effects of a specific mental disorder, we performed a sensitivity analysis that additionally adjusted for the presence of other mental disorders except the one we focused on. We also conducted subgroup analyses based on age (< 40, 40 to 64, and ≥ 65 years), sex, comorbidities, BMI (< 25, ≥ 25 kg/m^2^), DM duration (< 5 years, ≥ 5 years), insulin use, oral hypoglycemic agent use (< 3 agents, ≥ 3 agents), smoking (non/ex-smokers, and current smokers), drinking (non, mild drinkers, and heavy drinkers), regular exercise, and income (top 80% and bottom 20%). Statistical significance was set at P < 0.05. All statistical analyses were performed using the SAS statistical software (version 9.4; SAS Institute, Cary, NC, USA).

## Results

**Fig. 1:**
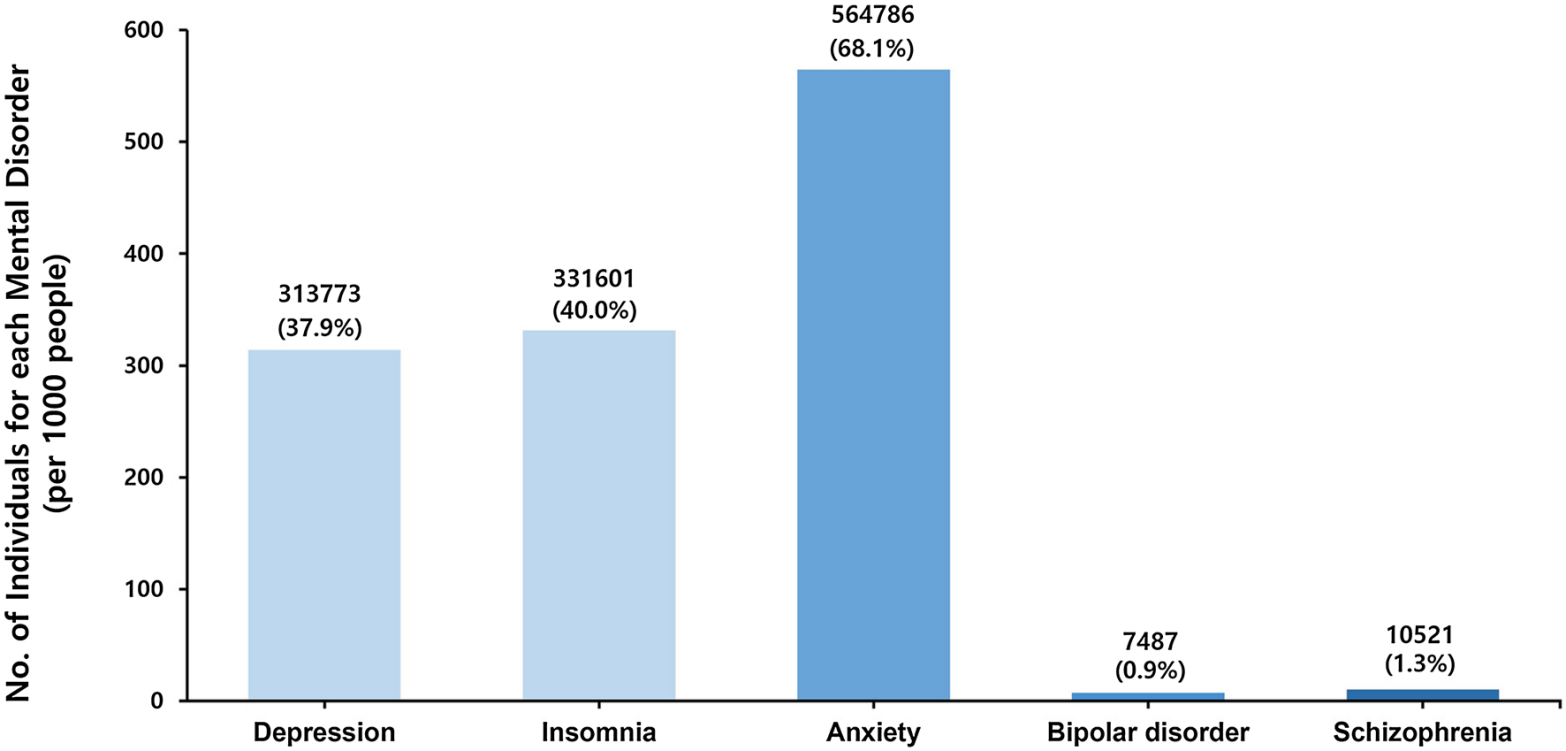
Number of individuals for each mental disorder in diabetic patients with mental disorder

A total of 2,512,690 patients with diabetes (mean age 57.2 ± 12.3 years, 60.1% men) were studied. The baseline characteristics of the two groups according to the presence or absence of mental disorders are shown in Table 1. Among the five mental disorders, anxiety (68.1%) was the most common followed by insomnia (40.0%) (Fig. 1). Patients with diabetes who had mental disorders were older and more likely to be women, compared with those without mental disorders. The proportion of patients with a diabetes duration of > 5 years was higher in patients with mental disorders, as was that of those prescribed insulin or ≥ 3 oral hypoglycemic agents. Patients with mental disorders had more comorbidities, including hypertension, dyslipidemia, chronic kidney disease, heart failure, obstructive sleep apnea, and thyroid disease, than those without mental disorders. The proportion of current smokers and heavy drinkers was greater among patients without mental disorders (Table 1). Additional file 1: Table S2 presents the baseline characteristics and demographic features of each group of patients with each mental disorder.

**Table 1:** Baseline characteristics

	Total	Mental disorder		
	Patients with/without mental disorder	Patients without mental disorder	Patients with mental disorder	
	n = 2,512,690	n = 1,683,761	n = 828,929	p-value
Age, years				< 0.001
≥ 65	736,231 (29.3%)	385,943 (22.9%)	350,288 (42.3%)	
Mean ± SD	57.17 ± 12.31	55.01 ± 12.22	61.58 ± 11.28	< 0.001
Sex				< 0.001
Male	1,510,711 (60.1%)	1,151,840 (68.4%)	358,871 (43.3%)	
Female	1,001,979 (39.9%)	531,921 (31.6%)	470,058 (56.7%)	
BMI (kg/m2)	25.07 ± 3.40	25.12 ± 3.40	24.96 ± 3.41	< 0.001
Hypertension	1,410,844 (56.2%)	881,787 (52.4%)	529,057 (63.8%)	< 0.001
Dyslipidemia	1,045,270 (41.6%)	642,901 (38.2%)	402,369 (48.5%)	< 0.001
CKD	280,310 (11.2%)	153,078 (9.1%)	127,232 (15.4%)	< 0.001
Heart failure	39,721 (1.6%)	17,176 (1.0%)	22,545 (2.7%)	< 0.001
OSA	2,826 (0.1%)	1,653 (0.1%)	1,173 (0.1%)	< 0.001
Thyroid disease	211,866 (8.4%)	66,595 (4.0%)	66,888 (8.1%)	< 0.001
Diabetes duration (≥ 5 years)	361,572 (14.4%)	457,384 (27.2%)	312,981 (37.8%)	< 0.001
Insulin use	133,483 (5.3%)	109,243 (6.5%)	102,623 (12.4%)	< 0.001
OHA (≥ 3 agents)	770,365 (30.7%)	217,710 (12.9%)	143,862 (17.4%)	< 0.001
Fasting glucose (mg/dL)	145.06 ± 47.09	148.15 ± 47.73	138.78 ± 45.12	< 0.001
Smoking				< 0.001
Non	1,392,615 (55.4%)	829,788 (49.3%)	562,827 (67.9%)	
Ex	461,643 (18.4%)	340,201 (20.2%)	121,442 (14.7%)	
Current	658,432 (26.2%)	513,772 (30.5%)	144,660 (17.5%)	
Drinking*				< 0.001
Non	1,429,128 (56.9%)	839,737 (49.9%)	589,391 (71.1%)	
Mild	830,840 (33.1%)	643,984 (38.3%)	186,856 (22.5%)	
Heavy	252,722 (10.1%)	200,040 (11.9%)	52,682 (6.4%)	
Regular exercise†	517,797 (20.6%)	352,671 (21.0%)	165,126 (19.9%)	< 0.001
Low income‡	528,625 (21.0%)	346,965 (20.6%)	181,660 (21.9%)	< 0.001

Values are mean ± SD or n (%). *Alcohol consumption is denoted as the following: nondrinker (alcohol consumption 0 g), mild to moderate drinker (alcohol consumption > 0 g to < 30 g/day), and heavy drinker (alcohol consumption ≥ 30 g/day). †Regular exercise denotes performing > 30 min of moderate-intensity exercise (e.g. brisk pace walking, tennis doubles, or bicycling leisurely) ≥ 5 times a week or > 20 min of vigorous-intensity exercise (e.g. running, climbing, fast cycling, or aerobics) ≥ 3 times a week. ‡Low income denotes income belongs to lower 20% among the entire Korean population of subjects supported by the Medical Aid program

*BMI* body mass index, *CKD* chronic kidney disease, *OSA* obstructive sleep apnea, *OHA* oral hypoglycemic agents

### Incidence rates and risks of new-onset AF

**Fig. 2:**
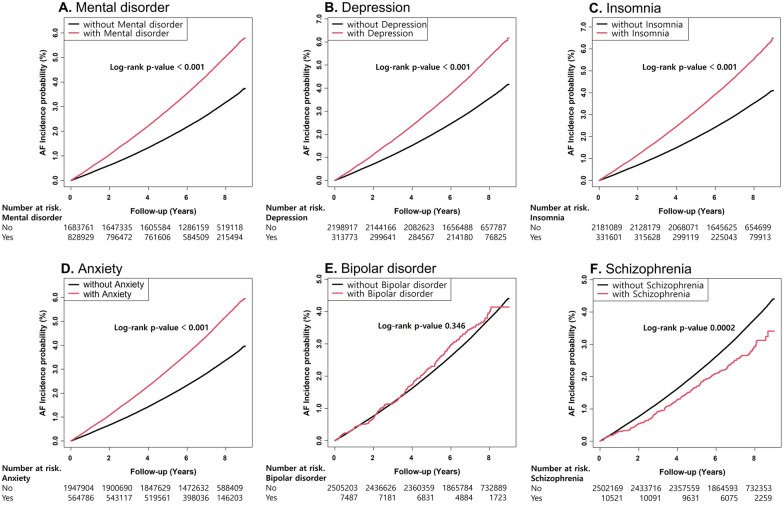
Kaplan-Meier curves for cumulative incidence of new-onset AF in patients with diabetes. AF, atrial fibrillation

The median follow-up duration was 7.1 years (interquartile range 5.9–8.1), and new-onset AF was diagnosed in 79,525 patients in the entire cohort. The crude incidence rate of new-onset AF was 4.66 per 1000 PY. Patients with diabetes who had mental disorders had a higher incidence of new-onset AF than those without mental disorder (6.27 per 1000 PY vs. 3.89 per 1000 PY, p < 0.001; Table 2). Figure 2 shows the Kaplan–Meier curves for the cumulative incidence of new-onset AF among patients with diabetes who had and did not have mental disorders. The cumulative incidence of new-onset AF was significantly higher in patients with diabetes who had depression, insomnia, or anxiety (all log-rank p < 0.001; Fig. 2). Among the mental disorders, patients with diabetes who had depression, insomnia, or anxiety had significantly higher risks for AF (unadjusted HR [95% CI]: 1.53 [1.50–1.56]; 1.61 [1.58–1.64]; 1.56 [1.54–1.59], respectively, all p < 0.001; Table 2).

**Table 2:** Multivariable Cox proportional hazards regression analysis of relationship between mental disorder and new-onset AF

	No. of individuals	AF	IR*	Model 1	p-value	Model 2	p-value	Model 3	p-value
Mental disorder									
No	1,683,761	45,002	3.89	1 (Ref.)	< 0.001	1 (Ref.)	< 0.001	1 (Ref.)	< 0.001
Yes	828,929	34,523	6.27	1.61 (1.60–1.64)		1.24 (1.22–1.25)		1.19 (1.17–1.21)	
Depression									
No	2,198,917	65,846	4.39	1 (Ref.)	< 0.001	1 (Ref.)	< 0.001	1 (Ref.)	< 0.001
Yes	313,773	13,679	6.66	1.53 (1.50–1.56)		1.20 (1.18–1.23)		1.15 (1.12–1.17)	
Insomnia									
No	2,181,089	64,575	4.33	1 (Ref.)	< 0.001	1 (Ref.)	< 0.001	1 (Ref.)	< 0.001
Yes	331,601	14,950	6.92	1.61 (1.58–1.64)		1.20 (1.18–1.22)		1.15 (1.13–1.18)	
Anxiety									
No	1,947,904	55,263	4.15	1 (Ref.)	< 0.001	1 (Ref.)	< 0.001	1 (Ref.)	< 0.001
Yes	564,786	24,262	6.46	1.56 (1.54–1.59)		1.23 (1.21–1.25)		1.19 (1.17–1.21)	
Bipolar disorder									
No	2,505,203	79,287	4.65	1 (Ref.)	0.33	1 (Ref.)	0.08	1 (Ref.)	0.16
Yes	7,487	238	4.91	1.07 (0.94–1.21)		1.12 (0.99–1.27)		1.10 (0.96–1.24)	
Schizophrenia									
No	2,502,169	79,284	4.66	1 (Ref.)	< 0.001	1 (Ref.)	0.10	1 (Ref.)	0.11
Yes	10,521	241	3.60	0.78 (0.69–0.89)		1.11 (0.98–1.26)		1.11 (0.98–1.26)	

Incidence and risk of new-onset AF according to the presence of mental disorder in patients with diabetes. Model 1: Cox proportional hazards model without adjustment. Model 2: Model 1 with adjustment for age and sex; Model 3: Model 2 with adjustment for BMI, low-income level, smoking status, alcohol consumption status, regular physical activity, hypertension, dyslipidemia, chronic kidney disease, heart failure, obstructive sleep apnea, thyroid disease, fasting glucose, DM duration, insulin use, and oral hypoglycemic agents

*AF* atrial fibrillation, *IR* Incidence rate, *CI* confidence interval, *HR* hazard ratio

^*^Incidence rates were calculated per 1,000 person-years

**Fig. 3:**
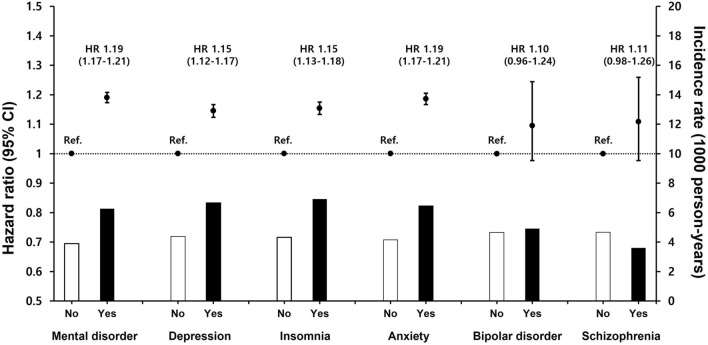
After multivariable adjustment, hazard ratios with 95% confidence intervals and incidence rate of new-onset AF for mental disorder. AF, atrial fibrillation; HR, hazard ratio; CI, confidence interval

After multivariable adjustment (Model 3), the risk for AF was consistently higher in patients with diabetes who had depression, insomnia, or anxiety (adjusted HR [95% CI]: 1.15 [1.12–1.17], 1.15 [1.13–1.18], 1.19 [1.67–1.21], all p < 0.001; Fig. 3). Bipolar disorder and schizophrenia were not significantly associated with AF risk.

### Sensitivity analysis

There are various types of mental disorders, and the coexistence of two or more mental disorders in an individual is possible. To assess the risk of AF for a specific mental disorder, a sensitivity analysis was performed using the multivariable Cox proportional hazard Model 3 with additional adjustment for mental disorders other than the one mental disorder of interest (Additional file 1: Table S3). The results of the sensitivity analysis were consistent with the main results. Patients with diabetes who had depression, insomnia, or anxiety had a significantly higher risk for AF (HR [95% CI]: 1.07 [1.05–1.09], 1.09 [1.07–1.12], 1.15 [1.13–1.17], all p < 0.001; Additional file 2: Figure S1).

### Subgroup analyses

The results of the subgroup analyses are presented in Additional file 1: Table S4. There was a significant interaction between mental disorders and age at risk of AF. Although a correlation between patients with diabetes who had mental disorders and a higher risk for AF was observed in all age groups, the relative risk of mental disorders for new-onset AF was accentuated in the younger age group (HR [95% CI]: 1.47 [1.22–1.75] in those aged < 40 years, 1.29 [1.26–1.32] in those aged 40 to 64 years, and 1.16 [1.14–1.18] in those aged ≥ 65 years; p for interaction < 0.001). In both the male and female patient subgroups, patients with mental disorders had a consistently higher risk for AF than those without mental disorders; the increase in relative risk was slightly higher in the male patient subgroup than in the female patient subgroup (p for interaction < 0.001). There was a significant interaction between the increase in relative risk for AF and subgroups stratified by hypertension, chronic kidney disease, and heart failure (all p for interaction < 0.05). In the subgroups of patients without hypertension, chronic kidney disease, and heart failure, those with mental disorders were consistently associated with a higher risk for AF, whereas in subgroups of patients with such comorbidities, the increase in relative risk of mental disorders was attenuated.

Subgroup analyses were performed for depression, insomnia, and anxiety (Additional file 1: Table S5). There was a significant interaction between each mental disorder and age-based subgroup for AF risk. The relative risk of depression, insomnia, or anxiety for new-onset AF was accentuated in the younger age subgroup. As subgroup analyses for mental disorders, there was a significant interaction between the relative risk increase of each mental disorder for new-onset AF and subgroups stratified by chronic kidney disease and heart failure (all p for interaction < 0.001). Patients with each mental disorder had a consistently higher risk for AF than those without each mental disorder in the subgroups without chronic kidney disease and heart failure.

## Discussion

In this large population-based study we used data from the NHID and found an association between mental disorders and the incidence of AF in patients with diabetes. Our study showed that a previous diagnosis of depression, insomnia, or anxiety was significantly associated with a higher risk for new-onset AF in patients with diabetes; however, a previous diagnosis of bipolar disorder and schizophrenia in patients with diabetes did not show statistically significant associations with the risk for AF. Second, the unfavorable effect of mental disorders on the risk for AF was more prominent in subgroups such as the younger groups and patients without hypertension, chronic kidney disease, or heart failure.

### Prevalence of mental disorders in patients with diabetes mellitus

Patients with chronic disorders are at an increased risk of developing mental disorders. Associations between mental disorders and chronic physical diseases such as cancer, heart disease, and diabetes have been reported [1, 27–29]. Especially anxiety and depression are two of the most common mental disorders worldwide [16]. The prevalence of depression is twice as common in patients with diabetes as in those without [30]. Indeed, the prevalence of depression in patients with diabetes ranges from 8.5% to 40.3% [31, 32] while that of anxiety ranges from 8.1% to 43.6% [33, 34]. However, the prevalence of bipolar disorder, schizophrenia, or insomnia in patients with DM is not well known. Our study, with its large sample size, also showed that anxiety, with a prevalence of 68.1%, was the most common mental disorder in patients with diabetes. The prevalence of depression was similar to that of insomnia. It is possible that the burden of caring for diabetes contributes to a decline in quality of life and leads to depression [35]. Symptom-related worries and illness-progression concerns in patients with diabetes could increase the likelihood of developing anxiety [36]. The coexistence of mental disorders and diabetes is reportedly associated with reduced quality of life and increases the economic burden on the health care system [37]. In patients with diabetes, vigilance is necessary for the prompt diagnosis and proactive management of mental disorders.

### Diabetes mellitus and atrial fibrillation as cardiovascular risk factors

As one of the most common chronic diseases, DM is a risk factor for stroke and heart disease like AF [5, 38] and is closely associated with CVDs [39]. CVDs are the most prevalent cause of morbidity and mortality in patients with DM [40]. The incidence of AF in patients with diabetes is reportedly 14.9%, and AF predicts a worse prognosis in patients with DM [41]. Patients with DM and new-onset AF have a higher risk of mortality than those with DM alone [42]. Therefore, greater awareness and improved detection of AF among patients with diabetes is important, given that AF onset may be asymptomatic and associated with adverse outcomes [43].

### Impact of mental disorders on the risk of atrial fibrillation in patients with diabetes mellitus

There are several studies that examined the relationship between mental disorders and new-onset AF. There is a multicenter cohort study of 6644 people aged 45 to 84 years without clinically recognized cardiovascular disease in the United States [21]. The study that assessed the relationships between depression with incident AF showed that depression increased the risk of new-onset AF by 34% [21]. A prospective cohort study showed that sleep disruption predicted incident AF in the Health eHeart Study and Cardiovascular Health Study populations over a median follow-up period of 11.6 years [44]. These results are consistent with our study results based on patients with DM. As for anxiety, anxiety could be an independent risk factor for AF [45], and a significant association between anxiety and AF risk was observed in our study. However, another study of 37,402 people in Norway revealed that there is no association between anxiety or depression and new-onset AF [22].

Regarding mental disorders and AF, imbalances in the autonomic nervous system, inflammatory processes, and renin-angiotensin-aldosterone system may play roles in the induction of new-onset AF [6, 21, 23, 24]. Stress situations such as depression and anxiety cause autonomic imbalance; catecholamine increases as sympathetic response increases, and parasympathetic response decreases [21, 24]. Fluctuations in autonomic tone and increased sympathetic activity by autonomic imbalance increase paroxysmal AF's incidence and produce an arrhythmogenic milieu in the atrium [24]. In stress situations such as depression and anxiety, the renin-angiotensin-aldosterone system is activated with increased sympathetic activity [21, 23]. The increased angiotensin II level causes atrial fibrosis and the increased left atrium as the consequence of atrial fibrosis are closely related to the development and maintenance of AF [21, 23, 24]. Inflammatory cytokines such as C-reactive protein, interleukin-6, and tumor necrosis factor-alpha, which increase under stress conditions, are also associated with the risk for incident AF [23, 24]. This process might affect more prominently in diabetic patients who are already in an inflammatory condition [46–48]. Sleep problems affect not only autonomic activity but also the hypothalamic–pituitary–adrenal axis [24, 49]. This results in neuroendocrine dysregulation and cortisol level increases [49, 50]. Consequently, increased cortisol level increases AF risk [51, 52].

There is little previous research on the association between bipolar disorder or schizophrenia and the risk of AF [6]. In patients with bipolar disorder or schizophrenia, their medical illnesses may be recognized and diagnosed late [2, 53, 54]. The possibility of underdiagnosis or under-recognition of AF due to communication impairment may affect our results.

Although we did not include information about psychotropic medications in the present study because of the inherent limitation of our dataset, psychotropic medications can also affect the arrhythmia risk [55]. Among various types of psychotropic drugs, the antipsychotic drug acts as a trigger factor leading to autonomic dysregulation and results in the occurrence of AF [56, 57]. Like chlorpromazine, clozapine, olanzapine, and risperidone, antipsychotic drugs may cause AF by alteration of autonomic tone [57].

On subgroup analysis, a significant interaction was observed between age and mental disorders and new-onset AF. Although a statistically significant association between mental disorders and AF was observed in all age groups, the relative risk increase of mental disorders for new-onset AF was more accentuated in the younger patients with diabetes. Age is well-known risk factor for AF and older patients are more likely to have AF risk factors than younger patients. Under these circumstances, the hazardous (or harmful) effect of mental disorders on new-onset AF might be more prominent in younger patients. Significant interactions were also observed between some comorbidities and mental disorders and new-onset AF. Mental disorders increased the risk for new-onset AF in patients with diabetes who did and did not have hypertension, CKD, or heart failure. However, the risk increase was more prominent in those without hypertension, CKD, or heart failure. Compared to patients with DM for more than 5 years, mental disorders increased the risk for AF more substantially in the subgroup of patients with DM for less than 5 years. Patients, who have hypertension, CKD, or heart failure, or have had DM for longer duration, could have comorbidities able to affect their risk for new-onset AF. Accordingly, mental disorders more significantly impacted the risk for new-onset AF in patients with diabetes who have had DM for shorter duration or did not have hypertension, CKD, or heart failure. Consequently, there was still a relative increased risk of mental disorders for new-onset AF in patients with previously lower risks for AF than in those who previously had higher AF risks.

### Study limitations

This study has some limitations. First, we used the Korean NHID, which comprises almost all East Asians. The results of this nationwide population-based cohort study may not be applicable to the global population. Nevertheless, our inclusion of a large number of patients is a strength. Second, we used ICD-10 codes based on insurance claims to define comorbidities and clinical outcomes. During diagnosis, coding inaccuracies might have occurred. However, the accuracy of AF diagnosis in the NHIS claims database has been validated in a previous study [58]. Lastly, although we adjusted several variables that were available in our dataset and could affect the risk of AF, still, there is a possibility of residual confounding factors by unmeasured factors. Also, we did not reflect the effect of newly diagnosed mental disorders after the index date in this study.

## Conclusions

Mental disorders, particularly depression, insomnia, and anxiety, are associated with an increased risk for AF in patients with diabetes. Greater awareness with a prompt diagnosis of AF should be considered for patients with both DM and mental disorders.

## Supplementary Information

**Additional file 1: Table S1.** Definition of covariates and outcomes. **Table S2.** Baseline characteristics of study patients with each mental disorder. **Table S3.** Sensitivity analysis. **Table S4.** Subgroup analysis of mental disorders. **Table S5.** Subgroup analysis of depression, insomnia, and anxiety.

**Additional file 2: Figure S1.** After sensitivity analysis, hazard ratios with 95% confidence intervals and incidence rate of new-onset AF for each mental disorder.

## Data Availability

All data generated or analyzed during this study are included in this article and its additional files. The datasets used in this study are available from the corresponding author on reasonable request.

## Abbreviations

AF: Atrial fibrillation
CVDs: Cardiovascular diseases
DM: Diabetes mellitus
NHID: National Health Information Database
NHIS: Korean National Health Insurance System
ICD-10: International Classification of Diseases 10th Revision
BMI: Body mass index
PY: Person-years
HRs: Hazard ratios
CIs: Confidence intervals

## References

[1] Momen NC, Plana-Ripoll O, Agerbo E, Benros ME, Borglum AD, Christensen MK (2020). Association between mental disorders and subsequent medical conditions. N Engl J Med.

[2] Rossom RC, Hooker SA, O'Connor PJ, Crain AL, Sperl-Hillen JM (2022). Cardiovascular risk for patients with and without schizophrenia, schizoaffective disorder, or bipolar disorder. J Am Heart Assoc.

[3] Peter RS, Jaensch A, Mons U, Schöttker B, Schmucker R, Koenig W (2021). Prognostic value of long-term trajectories of depression for incident diabetes mellitus in patients with stable coronary heart disease. Cardiovasc Diabetol.

[4] Macchi C, Favero C, Ceresa A, Vigna L, Conti DM, Pesatori AC (2020). Depression and cardiovascular risk-association among Beck Depression Inventory, PCSK9 levels and insulin resistance. Cardiovasc Diabetol.

[5] Hindricks G, Potpara T, Dagres N, Arbelo E, Bax JJ, Blomstrom-Lundqvist C (2021). 2020 ESC guidelines for the diagnosis and management of atrial fibrillation developed in collaboration with the European association for cardio-thoracic surgery (EACTS): the task force for the diagnosis and management of atrial fibrillation of the European society of cardiology (ESC) developed with the special contribution of the European heart rhythm association (EHRA) of the ESC. Eur Heart J.

[6] Yang HY, Huang JH, Lin YK, Hsu CY, Chen SA, Chen YJ (2014). Bipolar disorder and schizophrenia present different risks of atrial fibrillation: a nationwide population-based analysis. Acta Cardiol Sin.

[7] Fu Y, He W, Ma J, Wei B (2020). Relationship between psychological factors and atrial fibrillation: a meta-analysis and systematic review. Medicine (Baltimore).

[8] Teppo K, Jaakkola J, Lehto M, Biancari F, Airaksinen KEJ (2021). The impact of mental health conditions on oral anticoagulation therapy and outcomes in patients with atrial fibrillation: a systematic review and meta-analysis. Am J Prev Cardiol.

[9] Teppo K, Jaakkola J, Biancari F, Halminen O, Linna M, Putaala J (2022). Mental health conditions and bleeding events in patients with incident atrial fibrillation: a Finnish nationwide cohort study. Gen Hosp Psychiatry.

[10] Ling W, Huang Y, Huang YM, Fan RR, Sui Y, Zhao HL (2020). Global trend of diabetes mortality attributed to vascular complications, 2000–2016. Cardiovasc Diabetol.

[11] Patlolla SH, Lee HC, Noseworthy PA, Wysokinski WE, Hodge DO, Greene EL (2020). Impact of diabetes mellitus on stroke and survival in patients with atrial fibrillation. Am J Cardiol.

[12] Chan YH, Chuang C, Chan CC, Lee HF, Huang YC, Huang YT (2020). Glycemic status and risks of thromboembolism and major bleeding in patients with atrial fibrillation. Cardiovasc Diabetol.

[13] Bădescu SV, Tătaru C, Kobylinska L, Georgescu EL, Zahiu DM, Zăgrean AM (2016). The association between diabetes mellitus and depression. J Med Life.

[14] Koopman ADM, Beulens JW, Dijkstra T, Pouwer F, Bremmer MA, van Straten A (2020). Prevalence of insomnia (Symptoms) in T2D and association with metabolic parameters and glycemic control: meta-analysis. J Clin Endocrinol Metab.

[15] Choi EK (2020). Cardiovascular research using the Korean national health information database. Korean Circ J.

[16] Chaudhary R, Kumar P, Chopra A, Chabbra S, Singh P (2017). Comparative study of psychiatric manifestations among type I and type II diabetic patients. Indian J Psychol Med.

[17] Upthegrove R, Marwaha S, Birchwood M (2017). Depression and schizophrenia: cause, consequence, or trans-diagnostic issue?. Schizophr Bull.

[18] Achim AM, Maziade M, Raymond E, Olivier D, Mérette C, Roy MA (2011). How prevalent are anxiety disorders in schizophrenia? A meta-analysis and critical review on a significant association. Schizophr Bull.

[19] Pavlova B, Perlis RH, Mantere O, Sellgren CM, Isometsä E, Mitchell PB (2017). Prevalence of current anxiety disorders in people with bipolar disorder during euthymia: a meta-analysis. Psychol Med.

[20] Manning JS (2005). Burden of illness in bipolar depression. Prim Care Companion J Clin Psychiatry.

[21] Garg PK, O'Neal WT, Diez-Roux AV, Alonso A, Soliman EZ, Heckbert S (2019). Negative affect and risk of atrial fibrillation: MESA. J Am Heart Assoc.

[22] Feng T, Malmo V, Laugsand LE, Strand LB, Gustad LT, Ellekjær H (2020). Symptoms of anxiety and depression and risk of atrial fibrillation-The HUNT study. Int J Cardiol.

[23] Patel D, Mc Conkey ND, Sohaney R, Mc Neil A, Jedrzejczyk A, Armaganijan L (2013). A systematic review of depression and anxiety in patients with atrial fibrillation: the mind-heart link. Cardiovasc Psychiatry Neurol.

[24] Segan L, Prabhu S, Kalman JM, Kistler PM (2022). Atrial fibrillation and stress: A 2-way street?. JACC Clin Electrophysiol.

[25] Chao TF, Liu CJ, Tuan TC, Chen TJ, Hsieh MH, Lip GYH (2018). Lifetime risks, projected numbers, and adverse outcomes in Asian patients with atrial fibrillation: a report from the Taiwan nationwide AF cohort study. Chest.

[26] Lee SR, Choi EK, Han KD, Cha MJ, Oh S (2017). Trends in the incidence and prevalence of atrial fibrillation and estimated thromboembolic risk using the CHA2DS2-VASc score in the entire Korean population. Int J Cardiol.

[27] Balhara YP (2011). Diabetes and psychiatric disorders. Indian J Endocrinol Metab.

[28] Scott KM, de Jonge P, Alonso J, Viana MC, Liu Z, O'Neill S (2013). Associations between DSM-IV mental disorders and subsequent heart disease onset: beyond depression. Int J Cardiol.

[29] Scott KM, Lim C, Al-Hamzawi A, Alonso J, Bruffaerts R, Caldas-de-Almeida JM (2016). Association of mental disorders with subsequent chronic physical conditions: world mental health surveys from 17 countries. JAMA Psychiat.

[30] Roy T, Lloyd CE (2012). Epidemiology of depression and diabetes: a systematic review. J Affect Disord.

[31] Chaudhry R, Mishra P, Mishra J, Parminder S, Mishra BP (2010). Psychiatric morbidity among diabetic patients: a hospital-based study. Ind Psychiatry J.

[32] Niraula K, Kohrt BA, Flora MS, Thapa N, Mumu SJ, Pathak R (2013). Prevalence of depression and associated risk factors among persons with type-2 diabetes mellitus without a prior psychiatric history: a cross-sectional study in clinical settings in urban Nepal. BMC Psychiatry.

[33] Chaturvedi SK, Manche Gowda S, Ahmed HU, Alosaimi FD, Andreone N, Bobrov A (2019). More anxious than depressed: prevalence and correlates in a 15-nation study of anxiety disorders in people with type 2 diabetes mellitus. Gen Psychiatr.

[34] Al-Mohaimeed AA (2017). Prevalence and factors associated with anxiety and depression among type 2 diabetes in Qassim: a descriptive cross-sectional study. J Taibah Univ Med Sci.

[35] Katon WJ (2008). The comorbidity of diabetes mellitus and depression. Am J Med.

[36] Smith KJ, Béland M, Clyde M, Gariépy G, Pagé V, Badawi G (2013). Association of diabetes with anxiety: a systematic review and meta-analysis. J Psychosom Res.

[37] Ducat L, Philipson LH, Anderson BJ (2014). The mental health comorbidities of diabetes. JAMA.

[38] Huxley RR, Filion KB, Konety S, Alonso A (2011). Meta-analysis of cohort and case-control studies of type 2 diabetes mellitus and risk of atrial fibrillation. Am J Cardiol.

[39] Leon BM, Maddox TM (2015). Diabetes and cardiovascular disease: epidemiology, biological mechanisms, treatment recommendations and future research. World J Diabetes.

[40] Matheus AS, Tannus LR, Cobas RA, Palma CC, Negrato CA, Gomes MB (2013). Impact of diabetes on cardiovascular disease: an update. Int J Hypertens.

[41] De Sensi F, De Potter T, Cresti A, Severi S, Breithardt G (2015). Atrial fibrillation in patients with diabetes: molecular mechanisms and therapeutic perspectives. Cardiovasc Diagn Ther.

[42] Fatemi O, Yuriditsky E, Tsioufis C, Tsachris D, Morgan T, Basile J (2014). Impact of intensive glycemic control on the incidence of atrial fibrillation and associated cardiovascular outcomes in patients with type 2 diabetes mellitus (from the action to control cardiovascular risk in diabetes study). Am J Cardiol.

[43] Boriani G, Laroche C, Diemberger I, Fantecchi E, Popescu MI, Rasmussen LH (2015). Asymptomatic atrial fibrillation: clinical correlates, management, and outcomes in the EORP-AF Pilot general registry. Am J Med.

[44] Christensen MA, Dixit S, Dewland TA, Whitman IR, Nah G, Vittinghoff E (2018). Sleep characteristics that predict atrial fibrillation. Heart Rhythm.

[45] Severino P, Mariani MV, Maraone A, Piro A, Ceccacci A, Tarsitani L (2019). Triggers for atrial fibrillation: the role of anxiety. Cardiol Res Pract.

[46] Couzin-Frankel J (2010). Inflammation bares a dark side. Science.

[47] Donath MY, Shoelson SE (2011). Type 2 diabetes as an inflammatory disease. Nat Rev Immunol.

[48] Karam BS, Chavez-Moreno A, Koh W, Akar JG, Akar FG (2017). Oxidative stress and inflammation as central mediators of atrial fibrillation in obesity and diabetes. Cardiovasc Diabetol.

[49] Hirotsu C, Tufik S, Andersen ML (2015). Interactions between sleep, stress, and metabolism: from physiological to pathological conditions. Sleep Sci.

[50] Song HT, Sun XY, Yang TS, Zhang LY, Yang JL, Bai J (2015). Effects of sleep deprivation on serum cortisol level and mental health in servicemen. Int J Psychophysiol.

[51] Aguilar M, Rose RA, Takawale A, Nattel S, Reilly S (2021). New aspects of endocrine control of atrial fibrillation and possibilities for clinical translation. Cardiovasc Res.

[52] Larsson SC, Lee WH, Burgess S, Allara E (2021). Plasma cortisol and risk of atrial fibrillation: a Mendelian randomization study. J Clin Endocrinol Metab.

[53] Carney CP, Jones L, Woolson RF (2006). Medical comorbidity in women and men with schizophrenia: a population-based controlled study. J Gen Intern Med.

[54] Leboyer M, Kupfer DJ (2010). Bipolar disorder: new perspectives in health care and prevention. J Clin Psychiatry.

[55] Fanoe S, Kristensen D, Fink-Jensen A, Jensen HK, Toft E, Nielsen J (2014). Risk of arrhythmia induced by psychotropic medications: a proposal for clinical management. Eur Heart J.

[56] Iwamoto Y, Kawanishi C, Kishida I, Furuno T, Fujibayashi M, Ishii C (2012). Dose-dependent effect of antipsychotic drugs on autonomic nervous system activity in schizophrenia. BMC Psychiatry.

[57] Tisdale JE, Chung MK, Campbell KB, Hammadah M, Joglar JA, Leclerc J (2020). Drug-induced arrhythmias: a scientific statement from the American heart association. Circulation.

[58] Lee SS, Ae Kong K, Kim D, Lim YM, Yang PS, Yi JE (2017). Clinical implication of an impaired fasting glucose and prehypertension related to new onset atrial fibrillation in a healthy Asian population without underlying disease: a nationwide cohort study in Korea. Eur Heart J.

