# Supplementary material for: Impact of mental disorders on the risk of atrial fibrillation in patients with diabetes mellitus: a nationwide population-based study
Source: Cardiovasc Diabetol. 2022 Nov 17;21:251. doi: 10.1186/s12933-022-01682-7 (PMC9673441; doi:10.1186/s12933-022-01682-7)
Supplement: Supplementary file 1 — Additional file 1: Table S1. Definition of covariates and outcomes. Table S2. Baseline characteristics of study patients with each mental disorder. Table S3. Sensitivity analysis. Table S4. Subgroup analysis of mental disorders. Table S5. Subgroup analysis of depression, insomnia, and anxiety. [file 12933_2022_1682_MOESM1_ESM.docx]

**Additional file 1. Additional Material.**

**I. Additional file Tables**

Table S1. Definition of covariates and outcomes

Table S2. Baseline characteristics of study patients with each mental disorder

Table S3. Sensitivity analysis

Table S4. Subgroup analysis of mental disorders

Table S5. Subgroup analysis of depression, insomnia, and anxiety

**II. Additional file Figure Legends**

Figure S1. After sensitivity analysis, hazard ratios with 95% confidence intervals and incidence rate of new-onset AF for each mental disorder.

**I. Additional file Tables**

**Table S1. Definition of covariates and outcomes**

| **Diagnosis** | **ICD-10-CM code and definition** | **Diagnostic definition** |
| --- | --- | --- |
| **Inclusion** |  |  |
| **Diabetes mellitus** | E11-E14; and minimum 1 prescription of anti-diabetic drugs (sulfonylureas, metformin, meglitinides, thiazolidinediones, dipeptidyl peptidase-4 inhibitors, α-glucosidase inhibitors, and insulin). | Admission≥1 or outpatient department≥1 |
|  | Or fasting glucose level ≥ 126 mg/dL | Index health examination |
| **Depression** | F32-33 | Admission≥1 or outpatient department≥1 |
| **Insomnia** | F51, G47 | Admission≥1 or outpatient department≥1 |
| **Anxiety** | F40-41 | Admission≥1 or outpatient department≥1 |
| **Bipolar disorder** | F30-31 | Admission≥1 or outpatient department≥1 |
| **Schizophrenia** | F20 | Admission≥1 or outpatient department≥1 |
| **Outcome** |  |  |
| **Atrial fibrillation** | I48.0-48.4, I48.9 | Admission≥1 or outpatient department≥2 |
| **Comorbidities** |  |  |
| **Hypertension** | I10-I13, I15; and minimum 1 prescription of anti-hypertensive drug (thiazide, loop diuretics, aldosterone antagonist, alpha-/beta-blocker, calcium-channel blocker, angiotensin-converting enzyme inhibitor, angiotensin II receptor blocker). | Admission≥1 or outpatient department≥1 |
|  | Or systolic/diastolic blood pressure ≥ 140/90 mmHg | Index health examination |
| **Dyslipidemia** | E78 | Admission or outpatient department≥1 |
|  | Or Total cholesterol ≥ 240 mg/dL | Index health examination |
| **Chronic kidney disease** | Estimated glomerular filtration rate <60 ml/min/1.73m^2^ | Index health examination |
| **Heart failure** | I50 | Admission or outpatient department≥1 |
| **Obstructive sleep apnea** | G473 | Admission or outpatient department≥1 |
| **Thyroid disease** | E05 or E03 | Admission or outpatient department≥1 |
| **Definitions of lifestyle behavior based on the response of health examination questionnaire (Health examination before new-onset AF diagnosis)** | | |
| **Alcohol consumption** |  |  |
| Non-drinker | Alcohol consumption 0g |  |
| Mild to moderate drinker | Alcohol consumption > 0g to < 30g per day |  |
| Heavy drinker | Alcohol consumption ≥ 30g per day |  |
| **Smoking** |  |  |
| Ex-smoker | Ex-smoker at the 1st examination and sustaining non-smoking till the 2nd examination |  |
| Current smoker | Current smoker at the 2nd examination regardless of the smoking status at the 1st examination. |  |
| **Physical Exercise** |  |  |
| Regular exercise | Performing >30 minutes of moderate-intensity exercise (e.g. brisk pace walking, tennis doubles, or bicycling leisurely) ≥ 5 times a week or > 20 minutes of vigorous-intensity exercise (e.g. running, climbing, fast cycling, or aerobics) ≥ 3 times a week. |  |
| **Information of income** |  |  |
| **Low income** | Income belongs to lower 20% among the entire Korean population and supported by the Medical Aid program. |  |
| **Health checkup data** |  |  |
| Body mass index | Weight in kilograms divided by the square of height in meters (kg/m2) |  |

Abbreviations; ICD-10, 10^th^ revision of the international classification of disease; CM, clinical modification.

**Table S2. Baseline characteristics of study patients with each mental disorder**

|  | **Depression** | | | **Insomnia** | | | **Anxiety** | | | **Bipolar disorder** | | | **Schizophrenia** | | |
| --- | --- | --- | --- | --- | --- | --- | --- | --- | --- | --- | --- | --- | --- | --- | --- |
|  | No | Yes |  | No | Yes | p-value | No | Yes | p-value | No | Yes | p-value | No | Yes | p-value |
|  | 2198917 | 313773 |  | 2181089 | 331601 |  | 1947904 | 564786 |  | 2505203 | 7487 |  | 2502169 | 10521 |  |
| **Depression** | - | - | - | 194330  (8.9%) | 119443  (36.0%) | p<0.001 | 135594  (7.0%) | 178179  (31.6%) | p<0.001 | 309243  (12.3%) | 4530  (60.5%) | p<0.001 | 309396  (12.4%) | 4377  (41.6%) | p<0.001 |
| **Insomnia** | 212158  (9.7%) | 119443  (38.1%) | p<0.001 | - | - | - | 157449  (8.1%) | 174152  (30.8%) | p<0.001 | 327904  (13.1%) | 3697  (49.4%) | p<0.001 | 327895  (13.1%) | 3706  (35.2%) | p<0.001 |
| **Anxiety** | 386607  (17.6%) | 178179  (56.8%) | p<0.001 | 390634  (17.9%) | 174152  (52.5%) | p<0.001 | - | - | - | 560666  (22.4%) | 4120  (55.0%) | p<0.001 | 560664  (22.4%) | 4122  (39.2%) | p<0.001 |
| **Bipolar disorder** | 2957  (0.1%) | 4530  (1.4%) | p<0.001 | 3790  (0.2%) | 3697  (1.1%) | p<0.001 | 3367  (0.2%) | 4120  (0.7%) | p<0.001 | - | - | - | 6111  (0.2%) | 1376  (13.1%) | p<0.001 |
| **Schizophrenia** | 6144  (0.3%) | 4377  (1.4%) | p<0.001 | 6815  (0.3%) | 3706  (1.1%) | p<0.001 | 6399  (0.3%) | 4122  (0.7%) | p<0.001 | 9145  (0.4%) | 1376  (18.4%) | p<0.001 | - | - | - |
| **Age, years** |  |  | p<0.001 |  |  | p<0.001 |  |  | p<0.001 |  |  | p<0.001 |  |  | p<0.001 |
| **<40** | 188701  (8.6%) | 6807  (2.2%) |  | 188812  (8.7%) | 6696  (2.0%) |  | 181129  (9.3%) | 14379  (2.6%) |  | 195141  (7.8%) | 367  (4.9%) |  | 194851  (7.8%) | 657  (6.2%) |  |
| **40-64** | 1414840  (64.3%) | 166111  (52.9%) |  | 1412989  (64.8%) | 167962  (50.7%) |  | 1275193  (65.5%) | 305758  (54.1%) |  | 1575974  (62.9%) | 4977  (66.5%) |  | 1572829  (62.9%) | 8122  (77.2%) |  |
| **≥65** | 595376  (27.1%) | 140855  (44.9%) |  | 579288  (26.6%) | 156943  (47.3%) |  | 491582  (25.2%) | 244649  (43.3%) |  | 734088  (29.3%) | 2143  (28.6%) |  | 734489  (29.4%) | 1742  (16.6%) |  |
| **Mean ± SD** | 56.43  ±12.31 | 62.37  ±11.01 | p<0.001 | 56.29  ±12.25 | 62.98  ±11.1 | p<0.001 | 55.81  ±12.3 | 61.88  ±11.15 | p<0.001 | 57.17  ±12.31 | 57.42  ±11.74 | 0.0829 | 57.19  ±12.32 | 53.38  ±10.93 | p<0.001 |
| **Sex** |  |  | p<0.001 |  |  | p<0.001 |  |  | p<0.001 |  |  | p<0.001 |  |  | p<0.001 |
| **Male** | 1384999  (63.0%) | 125712  (40.1%) |  | 1377535  (63.2%) | 133176  (40.2%) |  | 1278693  (65.6%) | 232018  (41.1%) |  | 1507163  (60.2%) | 3548  (47.4%) |  | 1505403  (60.2%) | 5308  (50.5%) |  |
| **Female** | 813918  (37.0%) | 188061  (59.9%) |  | 803554  (36.8%) | 198425  (59.8%) |  | 669211  (34.4%) | 332768  (58.9%) |  | 998040  (39.8%) | 3939  (52.6%) |  | 996766  (39.8%) | 5213  (49.6%) |  |
| **BMI (kg/m2)** | 25.09  ±3.39 | 24.91  ±3.45 | p<0.001 | 25.1  ±3.4 | 24.83  ±3.44 | p<0.001 | 25.09  ±3.4 | 24.97  ±3.4 | p<0.001 | 25.06  ±3.4 | 25.74  ±3.81 | p<0.001 | 25.06  ±3.4 | 25.82  ±4.01 | p<0.001 |
| **Hypertension** | 1205978  (54.8%) | 204866  (65.3%) | p<0.001 | 1191579  (54.6%) | 219265  (66.1%) | p<0.001 | 1046693  (53.7%) | 364151  (64.5%) | p<0.001 | 1406976  (56.2%) | 3868  (51.7%) | p<0.001 | 1406275  (56.2%) | 4569  (43.4%) | p<0.001 |
| **Dyslipidemia** | 883774  (40.2%) | 161496  (51.5%) | p<0.001 | 880265  (40.4%) | 165005  (49.8%) | p<0.001 | 766477  (39.4%) | 278793  (49.4%) | p<0.001 | 1041749  (41.6%) | 3521  (47.0%) | p<0.001 | 1040812  (41.6%) | 4458  (42.4%) | 0.1071 |
| **CKD** | 226084  (10.3%) | 54226  (17.3%) | p<0.001 | 223731  (10.3%) | 56579  (17.1%) | p<0.001 | 192069  (9.9%) | 88241  (15.6%) | p<0.001 | 279345  (11.2%) | 965  (12.9%) | p<0.001 | 279172  (11.2%) | 1138  (10.8%) | 0.2679 |
| **Heart failure** | 29642  (1.4%) | 10079  (3.2%) | p<0.001 | 29058  (1.3%) | 10663  (3.2%) | p<0.001 | 23021  (1.2%) | 16700  (3.0%) | p<0.001 | 39570  (1.6%) | 151  (2.0%) | 0.0025 | 39561  (1.6%) | 160  (1.5%) | 0.6207 |
| **OSA** | 2266  (0.1%) | 560  (0.2%) | p<0.001 | 2280  (0.1%) | 546  (0.2%) | p<0.001 | 2025  (0.1%) | 801  (0.1%) | p<0.001 | 2805  (0.11%) | 21  (0.3%) | p<0.001 | 2805  (0.1%) | 21  (0.2%) | 0.0075 |
| **Thyroid disease** | 104080  (4.7%) | 29403  (9.4%) | p<0.001 | 104602  (4.8%) | 28881  (8.7%) | p<0.001 | 84872  (4.4%) | 48611  (8.6%) | p<0.001 | 132788  (5.3%) | 695  (9.3%) | p<0.001 | 132796  (5.3%) | 687  (6.5%) | p<0.001 |
| **Diabetes duration (≥5 years)** | 641809  (29.2%) | 128556  (41.0%) | p<0.001 | 638897  (29.3%) | 131468  (39.7%) | p<0.001 | 560082  (28.8%) | 210283  (37.2%) | p<0.001 | 767794  (30.7%) | 2571  (34.3%) | p<0.001 | 767339  (30.7%) | 3026  (28.8%) | p<0.001 |
| **Insulin use** | 163906  (7.5%) | 47960  (15.3%) | p<0.001 | 165514  (7.6%) | 46352  (14.0%) | p<0.001 | 142249  (7.3%) | 69617  (12.3%) | p<0.001 | 210899  (8.4%) | 967  (12.9%) | p<0.001 | 210831  (8.4%) | 1035  (9.8%) | p<0.001 |
| **OHA (≥3 agents)** | 303723  (13.8%) | 57849  (18.4%) | p<0.001 | 301733  (13.8%) | 59839  (18.1%) | p<0.001 | 264635  (13.6%) | 96937  (17.2%) | p<0.001 | 360262  (14.4%) | 1310  (17.5%) | p<0.001 | 359843  (14.4%) | 1729  (16.4%) | p<0.001 |
| **Fasting glucose (mg/dL)** | 146.14  ±47.13 | 137.51  ±46.09 | p<0.001 | 146.22  ±47.23 | 137.4  ±45.42 | p<0.001 | 147.11  ±47.65 | 137.99  ±44.37 | p<0.001 | 145.07  ±47.08 | 141.94  ±50.29 | p<0.001 | 145.06  ±47.05 | 145.66  ±55.33 | 0.1869 |
| **Smoking** |  |  | p<0.001 |  |  | p<0.001 |  |  | p<0.001 |  |  | p<0.001 |  |  | p<0.001 |
| **Non** | 1173372  (53.4%) | 219243  (69.9%) |  | 1162426  (53.3%) | 230189  (69.4%) |  | 999305  (51.3%) | 393310  (69.6%) |  | 1388022  (55.4%) | 4593  (61.4%) |  | 1386258  (55.4%) | 6357  (60.4%) |  |
| **Ex** | 418207  (19.0%) | 43436  (13.8%) |  | 416540  (19.1%) | 45103  (13.6%) |  | 381829  (19.6%) | 79814  (14.1%) |  | 460724  (18.4%) | 919  (12.3%) |  | 460616  (18.4%) | 1027  (9.8%) |  |
| **Current** | 607338  (27.6%) | 51094  (16.3%) |  | 602123  (27.6%) | 56309  (17.0%) |  | 566770  (29.1%) | 91662  (16.2%) |  | 656457  (26.2%) | 1975  (26.4%) |  | 655295  (26.2%) | 3137  (29.8%) |  |
| **Drinking*** |  |  | p<0.001 |  |  | p<0.001 |  |  | p<0.001 |  |  | p<0.001 |  |  | p<0.001 |
| **Non** | 1194142  (54.3%) | 234986  (74.9%) |  | 1184508  (54.3%) | 244620  (73.8%) |  | 1019497  (52.3%) | 409631  (72.5%) |  | 1423522  (56.8%) | 5606  (74.9%) |  | 1420632  (56.8%) | 8496  (80.8%) |  |
| **Mild** | 768958  (35.0%) | 61882  (19.7%) |  | 763084  (35.0%) | 67756  (20.4%) |  | 709159  (36.4%) | 121681  (21.5%) |  | 829310  (33.1%) | 1530  (20.4%) |  | 829171  (33.1%) | 1669  (15.9%) |  |
| **Heavy** | 235817  (10.7%) | 16905  (5.4%) |  | 233497  (10.7%) | 19225  (5.8%) |  | 219248  (11.3%) | 33474  (5.9%) |  | 252371  (10.1%) | 351  (4.7%) |  | 252366  (10.1%) | 356  (3.4%) |  |
| **Regular exercise†** | 457026  (20.8%) | 60771  (19.4%) | p<0.001 | 452381  (20.7%) | 65416  (19.7%) | p<0.001 | 406005  (20.8%) | 111792  (19.8%) | p<0.001 | 516395  (20.6%) | 1402  (18.7%) | p<0.001 | 516251  (20.6%) | 1546  (14.7%) | p<0.001 |
| **Low income‡** | 459607  (20.9%) | 69018  (22.0%) | p<0.001 | 453708  (20.8%) | 74917  (22.6%) | p<0.001 | 406108  (20.9%) | 122517  (21.7%) | p<0.001 | 526565  (21.0%) | 2060  (27.5%) | p<0.001 | 524198  (21.0%) | 4427  (42.1%) | p<0.001 |

Values are mean ± SD or n (%). *Alcohol consumption is denoted as the following: nondrinker (alcohol consumption 0 g), mild to moderate drinker (alcohol consumption >0 g to <30 g/day), and heavy drinker (alcohol consumption ≥ 30 g/day). †Regular exercise denotes performing >30 minutes of moderate-intensity exercise (e.g. brisk pace walking, tennis doubles, or bicycling leisurely) ≥ 5 times a week or > 20 minutes of vigorous-intensity exercise (e.g. running, climbing, fast cycling, or aerobics) ≥ 3 times a week. ‡Low income denotes income belongs to lower 20% among the entire Korean population of subjects supported by the Medical Aid program.

Abbreviations; BMI, body mass index; CKD, chronic kidney disease; OSA, obstructive sleep apnea; OHA, oral hypoglycemic agents.

**Table S3. Sensitivity analysis**

|  | **No. of**  **individuals** | **AF** | **IR*** | **Adjusted HR (95% CI)** | **p-value** |
| --- | --- | --- | --- | --- | --- |
| **Depression** |  |  |  |  |  |
| **No** | 2,198,917 | 65,846 | 4.39 | 1 (Ref.) | <0.001 |
| **Yes** | 313,773 | 13,679 | 6.66 | 1.07 (1.05-1.09) |  |
| **Insomnia** |  |  |  |  |  |
| **No** | 2,181,089 | 64,575 | 4.33 | 1 (Ref.) | <0.001 |
| **Yes** | 331,601 | 14,950 | 6.92 | 1.09 (1.07-1.12) |  |
| **Anxiety** |  |  |  |  |  |
| **No** | 1,947,904 | 55,263 | 4.15 | 1 (Ref.) | <0.001 |
| **Yes** | 564,786 | 24,262 | 6.46 | 1.15 (1.13-1.17) |  |
| **Bipolar disorder** |  |  |  |  |  |
| **No** | 2,505,203 | 79,287 | 4.65 | 1 (Ref.) | 0.78 |
| **Yes** | 7,487 | 238 | 4.91 | 0.98 (0.86-1.12) |  |
| **Schizophrenia** |  |  |  |  |  |
| **No** | 2,502,169 | 79,284 | 4.66 | 1 (Ref.) | 0.54 |
| **Yes** | 10,521 | 241 | 3.60 | 1.04 (0.92-1.18) |  |

*Incidence rates were calculated per 1,000 person-years.

Adjusted for age, sex, BMI, low-income level, smoking status, alcohol consumption status, regular physical activity, hypertension, dyslipidemia, chronic kidney disease, heart failure, obstructive sleep apnea, thyroid disease, fasting glucose, DM duration, insulin use, oral hypoglycemic agents and other mental disorders except interested mental disorder type.

Abbreviations; AF, atrial fibrillation; IR, Incidence rate; CI, confidence interval; HR, hazard ratio.

**Table S4. Subgroup analysis of mental disorders**

|  | **Mental disorder** | **No** | **AF** | **IR*** | **HR (95% CI)** |
| --- | --- | --- | --- | --- | --- |
| **Age, years** |  |  |  |  | p for interaction <0.001 |
| **<40** | No | 172,460 | 753 | 0.63 | 1 (Ref.) |
|  | Yes | 23,048 | 141 | 0.89 | 1.47 (1.22-1.75) |
| **40-64** | No | 1125,358 | 21,550 | 2.75 | 1 (Ref.) |
|  | Yes | 455,593 | 11,605 | 3.72 | 1.29 (1.26-1.32) |
| **≥65** | No | 385,943 | 22,699 | 8.98 | 1 (Ref.) |
|  | Yes | 350,288 | 22,777 | 10.24 | 1.16 (1.14-1.18) |
| **Sex** |  |  |  |  | p for interaction = 0.02 |
| **Male** | No | 1,151,840 | 31,716 | 4.03 | 1 (Ref.) |
|  | Yes | 358,871 | 16,023 | 6.89 | 1.19 (1.17-1.21) |
| **Female** | No | 531,921 | 13,286 | 3.59 | 1 (Ref.) |
|  | Yes | 470,058 | 18,500 | 5.82 | 1.23 (1.21-1.26) |
| **BMI (kg/m2)** |  |  |  |  | p for interaction = 0.88 |
| **<25** | No | 853,425 | 22,165 | 3.80 | 1 (Ref.) |
|  | Yes | 435,753 | 17,391 | 6.10 | 1.21 (1.18-1.23) |
| **≥25** | No | 830,336 | 22,837 | 3.98 | 1 (Ref.) |
|  | Yes | 393,176 | 17,132 | 6.46 | 1.21 (1.18-1.23) |
| **Hypertension** |  |  |  |  | p for interaction = 0.04 |
| **No** | No | 801,974 | 13,187 | 2.37 | 1 (Ref.) |
|  | Yes | 299,872 | 7,866 | 3.88 | 1.24 (1.21-1.28) |
| **Yes** | No | 881,787 | 31,815 | 5.29 | 1 (Ref.) |
|  | Yes | 529,057 | 26,657 | 7.66 | 1.20 (1.18-1.22) |
| **Dyslipidemia** |  |  |  |  | p for interaction = 0.25 |
| **No** | No | 1,040,860 | 27,090 | 3.80 | 1 (Ref.) |
|  | Yes | 426,560 | 17,588 | 6.25 | 1.20 (1.18-1.22) |
| **Yes** | No | 642,901 | 17,912 | 4.04 | 1 (Ref.) |
|  | Yes | 402,369 | 16,935 | 6.29 | 1.22 (1.19-1.25) |
| **CKD** |  |  |  |  | p for interaction <0.001 |
| **No** | No | 1,530,683 | 36,871 | 3.50 | 1 (Ref.) |
|  | Yes | 701,697 | 26,047 | 5.54 | 1.23 (1.21-1.25) |
| **Yes** | No | 153,078 | 8,131 | 7.94 | 1 (Ref.) |
|  | Yes | 127,232 | 8,476 | 10.57 | 1.14 (1.11-1.18) |
| **Heart failure** |  |  |  |  | p for interaction <0.001 |
| **No** | No | 1,666,585 | 43,207 | 3.77 | 1 (Ref.) |
|  | Yes | 806,384 | 32,151 | 5.98 | 1.21 (1.19-1.23) |
| **Yes** | No | 17,176 | 1,795 | 17.22 | 1 (Ref.) |
|  | Yes | 22,545 | 2,372 | 17.94 | 0.94 (0.88-1.00) |
| **Diabetes duration (≥5 years)** |  |  |  |  | p for interaction <0.001 |
| **<5 years** | No | 1,226,377 | 27,620 | 3.28 | 1 (Ref.) |
|  | Yes | 515,948 | 19,073 | 5.55 | 1.25 (1.23-1.27) |
| **≥5 years** | No | 457,384 | 17,382 | 5.53 | 1 (Ref.) |
|  | Yes | 312,981 | 15,450 | 7.47 | 1.16 (1.13-1.18) |
| **Insulin use** |  |  |  |  | p for interaction = 0.33 |
| **No** | No | 1,574,518 | 40,251 | 3.71 | 1 (Ref.) |
|  | Yes | 726,306 | 28,620 | 5.89 | 1.21 (1.19-1.23) |
| **Yes** | No | 109,243 | 4,751 | 6.62 | 1 (Ref.) |
|  | Yes | 102,623 | 5,903 | 9.18 | 1.19 (1.14-1.23) |
| **OHA (≥3 agents)** |  |  |  |  | p for interaction = 0.34 |
| **<3** | No | 1,466,051 | 37,603 | 3.74 | 1 (Ref.) |
|  | Yes | 685,067 | 27,886 | 6.14 | 1.21 (1.19-1.23) |
| **≥3** | No | 217,710 | 7,399 | 4.89 | 1 (Ref.) |
|  | Yes | 143,862 | 6,637 | 6.88 | 1.19 (1.15-1.23) |
| **Smoking** |  |  |  |  | p for interaction = 0.02 |
| **Non, Ex** | No | 1,169,989 | 33,764 | 4.19 | 1 (Ref.) |
|  | Yes | 684,269 | 29,276 | 6.41 | 1.20 (1.18-1.22) |
| **Current** | No | 513,772 | 11,238 | 3.21 | 1 (Ref.) |
|  | Yes | 144,660 | 5,247 | 5.61 | 1.25 (1.21-1.29) |
| **Drinking** |  |  |  |  | p for interaction = 0.94 |
| **Non, Mild** | No | 1,483,721 | 39,669 | 3.89 | 1 (Ref.) |
|  | Yes | 776,247 | 32,426 | 6.29 | 1.21 (1.19-1.23) |
| **Heavy** | No | 200,040 | 5,333 | 3.91 | 1 (Ref.) |
|  | Yes | 52,682 | 2,097 | 6.06 | 1.21 (1.15-1.27) |
| **Regular exercise** |  |  |  |  | p for interaction = 0.72 |
| **No** | No | 1,331,090 | 35,229 | 3.87 | 1 (Ref.) |
|  | Yes | 663,803 | 28,082 | 6.41 | 1.21 (1.19-1.23) |
| **Yes** | No | 352,671 | 9,773 | 3.98 | 1 (Ref.) |
|  | Yes | 165,126 | 6,441 | 5.74 | 1.20 (1.17-1.24) |
| **Income** |  |  |  |  | p for interaction = 0.35 |
| **Top 80%** | No | 1,336,796 | 35,486 | 3.86 | 1 (Ref.) |
|  | Yes | 647,269 | 27,378 | 6.34 | 1.21 (1.19-1.23) |
| **bottom 20%** | No | 346,965 | 9,516 | 4.02 | 1 (Ref.) |
|  | Yes | 181,660 | 7,145 | 6.03 | 1.22 (1.19-1.26) |

*Incidence rates were calculated per 1,000 person-years.

Adjusted for age, sex, BMI, low-income level, smoking status, alcohol consumption status, regular physical activity, hypertension, dyslipidemia, chronic kidney disease, heart failure, obstructive sleep apnea, thyroid disease, fasting glucose, DM duration, insulin use, and oral hypoglycemic agents.

Abbreviations; AF, atrial fibrillation; IR, Incidence rate; CI, confidence interval; HR, hazard ratio.

**Table S5. Subgroup analysis of depression, insomnia, and anxiety**

|  |  | **Depression** | | | | **Insomnia** | | | | **Anxiety** | | | |
| --- | --- | --- | --- | --- | --- | --- | --- | --- | --- | --- | --- | --- | --- |
|  | **Mental disorder** | **No** | **AF** | **IR*** | **Adjusted HR (95% CI)** | **No** | **AF** | **IR** | **Adjusted HR (95% CI)** | **No** | **AF** | **IR** | **Adjusted HR (95% CI)** |
| **Age, years** |  |  |  |  | P_int_ < 0.001 |  |  |  | P_int_ < 0.001 |  |  |  | P_int_ < 0.001 |
| **<40** | No | 188701 | 846 | 0.6434 | 1 (Ref.) | 188812 | 849 | 0.6453 | 1 (Ref.) | 181129 | 807 | 0.6391 | 1 (Ref.) |
|  | Yes | 6807 | 48 | 1.0386 | 1.66 (1.24-2.22) | 6696 | 45 | 0.9895 | 1.59 (1.18-2.15) | 14379 | 87 | 0.8828 | 1.41 (1.13-1.75) |
| **40-64** | No | 1414840 | 28641 | 2.9141 | 1 (Ref.) | 1412989 | 28584 | 2.9111 | 1 (Ref.) | 1275193 | 25195 | 2.8448 | 1 (Ref.) |
|  | Yes | 166111 | 4514 | 4.0077 | 1.29 (1.25-1.33) | 167962 | 4571 | 4.0242 | 1.30 (1.26-1.34) | 305758 | 7960 | 3.7934 | 1.28 (1.25-1.31) |
| **≥65** | No | 595376 | 36359 | 9.3889 | 1 (Ref.) | 579288 | 35142 | 9.3095 | 1 (Ref.) | 491582 | 29261 | 9.1547 | 1 (Ref.) |
|  | Yes | 140855 | 9117 | 10.346 | 1.11 (1.08-1.13) | 156943 | 10334 | 10.5567 | 1.12 (1.09-1.14) | 244649 | 16215 | 10.4109 | 1.17 (1.14-1.19) |
| **Sex** |  |  |  |  | P_int_ = 0.28 |  |  |  | P_int_ = 0.91 |  |  |  | P_int_ = 0.03 |
| **Male** | No | 1384999 | 41888 | 4.45915 | 1 (Ref.) | 1377535 | 41258 | 4.41063 | 1 (Ref.) | 1278693 | 37108 | 4.27198 | 1 (Ref.) |
|  | Yes | 125712 | 5851 | 7.33408 | 1.152 (1.12-1.18) | 133176 | 6481 | 7.74048 | 1.17 (1.14-1.20) | 232018 | 10631 | 7.06323 | 1.19 (1.16-1.21) |
| **Female** | No | 813918 | 23958 | 4.26118 | 1 (Ref.) | 803554 | 23317 | 4.19718 | 1 (Ref.) | 669211 | 18155 | 3.92196 | 1 (Ref.) |
|  | Yes | 188061 | 7828 | 6.23261 | 1.18 (1.15-1.21) | 198425 | 8469 | 6.40151 | 1.18 (1.15-1.20) | 332768 | 13631 | 6.0601 | 1.23 (1.2-1.26) |
| **BMI (kg/m2)** |  |  |  |  | P_int_ = 0.79 |  |  |  | P_int_ = 0.58 |  |  |  | P_int_ = 0.51 |
| **<25** | No | 1122424 | 32612 | 4.28824 | 1 (Ref.) | 1109817 | 31886 | 4.23391 | 1 (Ref.) | 993321 | 27439 | 4.07245 | 1 (Ref.) |
|  | Yes | 166754 | 6944 | 6.47546 | 1.17 (1.14-1.20) | 179361 | 7670 | 6.69149 | 1.17 (1.14-1.20) | 295857 | 12117 | 6.2471 | 1.20 (1.17-1.23) |
| **≥25** | No | 1076493 | 33234 | 4.48434 | 1 (Ref.) | 1071272 | 32689 | 4.4303 | 1 (Ref.) | 954583 | 27824 | 4.23003 | 1 (Ref.) |
|  | Yes | 147019 | 6735 | 6.86264 | 1.16 (1.13-1.19) | 152240 | 7280 | 7.17932 | 1.18 (1.15-1.21) | 268929 | 12145 | 6.69219 | 1.21 (1.19-1.24) |
| **Hypertension** |  |  |  |  | P_int_ = 0.06 |  |  |  | P_int_ = 0.08 |  |  |  | P_int_ = 0.06 |
| **No** | No | 992939 | 18038 | 2.63085 | 1 (Ref.) | 989510 | 17843 | 2.60979 | 1 (Ref.) | 901211 | 15590 | 2.50466 | 1 (Ref.) |
|  | Yes | 108907 | 3015 | 4.14982 | 1.20 (1.16-1.25) | 112336 | 3210 | 4.30346 | 1.21 (1.16-1.26) | 200635 | 5463 | 4.02143 | 1.24 (1.20-1.28) |
| **Yes** | No | 1205978 | 47808 | 5.85898 | 1 (Ref.) | 1191579 | 46732 | 5.78893 | 1 (Ref.) | 1046693 | 39673 | 5.5948 | 1 (Ref.) |
|  | Yes | 204866 | 10664 | 8.03484 | 1.15 (1.13-1.18) | 219265 | 11740 | 8.30067 | 1.16 (1.14-1.19) | 364151 | 18799 | 7.84615 | 1.20 (1.18-1.22) |
| **Dyslipidemia** |  |  |  |  | P_int_ = 0.92 |  |  |  | P_int_ =0.25 |  |  |  | P_int_ = 0.24 |
| **No** | No | 1315143 | 38100 | 4.25182 | 1 (Ref.) | 1300824 | 37237 | 4.19626 | 1 (Ref.) | 1181427 | 32548 | 4.03937 | 1 (Ref.) |
|  | Yes | 152277 | 6578 | 6.66479 | 1.16 (1.13-1.20) | 166596 | 7441 | 6.92836 | 1.16 (1.13-1.19) | 285993 | 12130 | 6.41748 | 1.20 (1.17-1.22) |
| **Yes** | No | 883774 | 27746 | 4.58215 | 1 (Ref.) | 880265 | 27338 | 4.52934 | 1 (Ref.) | 766477 | 22715 | 4.32029 | 1 (Ref.) |
|  | Yes | 161496 | 7101 | 6.65648 | 1.17 (1.14-1.20) | 165005 | 7509 | 6.91268 | 1.19 (1.16-1.22) | 278793 | 12132 | 6.50763 | 1.22 (1.19-1.25) |
| **CKD** |  |  |  |  | P_int_ < 0.001 |  |  |  | P_int_ < 0.001 |  |  |  | P_int_ < 0.001 |
| **No** | No | 1972833 | 52857 | 3.9082 | 1 (Ref.) | 1957358 | 51905 | 3.8645 | 1 (Ref.) | 1755835 | 44637 | 3.7052 | 1 (Ref.) |
|  | Yes | 259547 | 10061 | 5.8536 | 1.19 (1.16-1.21) | 275022 | 11013 | 6.0763 | 1.19 (1.17-1.22) | 476545 | 18281 | 5.7193 | 1.23 (1.21-1.25) |
| **Yes** | No | 226084 | 12989 | 8.7097 | 1 (Ref.) | 223731 | 12670 | 8.5694 | 1 (Ref.) | 192069 | 10626 | 8.3784 | 1 (Ref.) |
|  | Yes | 54226 | 3618 | 10.8005 | 1.10 (1.06-1.14) | 56579 | 3937 | 11.3199 | 1.12 (1.08-1.17) | 88241 | 5981 | 10.7178 | 1.14 (1.10-1.17) |
| **Heart failure** |  |  |  |  | P_int_ < 0.001 |  |  |  | P_int_ < 0.001 |  |  |  | P_int_ < 0.001 |
| **No** | No | 2169275 | 62662 | 4.223 | 1 (Ref.) | 2152031 | 61533 | 4.1761 | 1 (Ref.) | 1924883 | 52855 | 4.0111 | 1 (Ref.) |
|  | Yes | 303694 | 12696 | 6.3631 | 1.17 (1.15-1.20) | 320938 | 13825 | 6.5868 | 1.18 (1.16-1.20) | 548086 | 22503 | 6.1549 | 1.21 (1.19-1.23) |
| **Yes** | No | 29642 | 3184 | 17.8894 | 1 (Ref.) | 29058 | 3042 | 17.3716 | 1 (Ref.) | 23021 | 2408 | 17.4296 | 1 (Ref.) |
|  | Yes | 10079 | 983 | 16.8039 | 0.89 (0.83-0.96) | 10663 | 1125 | 18.3322 | 0.93 (0.87-1.00) | 16700 | 1759 | 17.8896 | 0.96 (0.91-1.03) |
| **Diabetes duration** |  |  |  |  | P_int_ < 0.001 |  |  |  | P_int_ < 0.001 |  |  |  | P_int_ < 0.001 |
| **<5 years** | No | 1557108 | 39539 | 3.7157 | 1 (Ref.) | 1542192 | 38577 | 3.65689 | 1 (Ref.) | 1387822 | 33137 | 3.48938 | 1 (Ref.) |
|  | Yes | 185217 | 7154 | 5.87574 | 1.20 (1.17-1.23) | 200133 | 8116 | 6.19791 | 1.21 (1.18-1.24) | 354503 | 13556 | 5.73901 | 1.24 (1.22-1.27) |
| **≥5 years** | No | 641809 | 26307 | 6.01297 | 1 (Ref.) | 638897 | 25998 | 5.96221 | 1 (Ref.) | 560082 | 22126 | 5.79381 | 1 (Ref.) |
|  | Yes | 128556 | 6525 | 7.80308 | 1.13 (1.10-,1.16) | 131468 | 6834 | 8.03262 | 1.13 (1.10-1.16) | 210283 | 10706 | 7.6892 | 1.16 (1.14-1.19) |
| **Insulin use** |  |  |  |  | P_int_ = 0.23 |  |  |  | P_int_ = 0.77 |  |  |  | P_int_ = 0.04 |
| **No** | No | 2035011 | 57987 | 4.15633 | 1 (Ref.) | 2015575 | 56733 | 4.10176 | 1 (Ref.) | 1805655 | 48714 | 3.93123 | 1 (Ref.) |
|  | Yes | 265813 | 10884 | 6.1939 | 1.17 (1.15-1.20) | 285249 | 12138 | 6.46567 | 1.17 (1.15-1.20) | 495169 | 20157 | 6.07661 | 1.22 (1.19-1.24) |
| **Yes** | No | 163906 | 7859 | 7.38193 | 1 (Ref.) | 165514 | 7842 | 7.27314 | 1 (Ref.) | 142249 | 6549 | 7.08851 | 1 (Ref.) |
|  | Yes | 47960 | 2795 | 9.42526 | 1.14 (1.09-1.19) | 46352 | 2812 | 9.93793 | 1.18 (1.13-1.23) | 69617 | 4105 | 9.38757 | 1.16 (1.12-1.21) |
| **OHA** |  |  |  |  | P_int_ = 0.93 |  |  |  | P_int_ = 0.30 |  |  |  | P_int_ = 0.98 |
| **<3** | No | 1895194 | 54579 | 4.22421 | 1 (Ref.) | 1879356 | 53453 | 4.16769 | 1 (Ref.) | 1683269 | 45882 | 3.99384 | 1 (Ref.) |
|  | Yes | 255924 | 10910 | 6.52486 | 1.16 (1.14-1.19) | 271762 | 12036 | 6.81143 | 1.18 (1.16-1.20) | 467849 | 19607 | 6.31586 | 1.21 (1.19-1.23) |
| **≥3** | No | 303723 | 11267 | 5.37656 | 1 (Ref.) | 301733 | 11122 | 5.33674 | 1 (Ref.) | 264635 | 9381 | 5.13395 | 1 (Ref.) |
|  | Yes | 57849 | 2769 | 7.25455 | 1.16 (1.12-1.21) | 59839 | 2914 | 7.4105 | 1.15 (1.11-1.20) | 96937 | 4655 | 7.16133 | 1.21 (1.16-1.25) |
| **Smoking** |  |  |  |  | P_int_ = 0.002 |  |  |  | P_int_ = 0.03 |  |  |  | P_int_ = 0.001 |
| **Non, Ex** | No | 1591579 | 51326 | 4.70548 | 1 (Ref.) | 1578966 | 50288 | 4.64255 | 1 (Ref.) | 1381134 | 42266 | 4.46088 | 1 (Ref.) |
|  | Yes | 262679 | 11714 | 6.77115 | 1.15 (1.13-1.18) | 275292 | 12752 | 7.06194 | 1.16 (1.14-1.19) | 473124 | 20774 | 6.56805 | 1.19 (1.17-1.21) |
| **Current** | No | 607338 | 14520 | 3.53423 | 1 (Ref.) | 602123 | 14287 | 3.50374 | 1 (Ref.) | 566770 | 12997 | 3.38409 | 1 (Ref.) |
|  | Yes | 51094 | 1965 | 6.0691 | 1.25 (1.19-1.31) | 56309 | 2198 | 6.19993 | 1.23 (1.18-1.29) | 91662 | 3488 | 5.8965 | 1.28 (1.23-1.32) |
| **Drinking** |  |  |  |  | P_int_ = 0.15 |  |  |  | P_int_ = 0.87 |  |  |  | P_int_ = 0.96 |
| **Non, Mild** | No | 1963100 | 59132 | 4.40813 | 1 (Ref.) | 1947592 | 57938 | 4.34909 | 1 (Ref.) | 1728656 | 49189 | 4.1598 | 1 (Ref.) |
|  | Yes | 296868 | 12963 | 6.66619 | 1.16 (1.14-1.18) | 312376 | 14157 | 6.94982 | 1.17 (1.15-1.20) | 531312 | 22906 | 6.48154 | 1.21 (1.19-1.23) |
| **Heavy** | No | 235817 | 6714 | 4.19153 | 1 (Ref.) | 233497 | 6637 | 4.18014 | 1 (Ref.) | 219248 | 6074 | 4.07492 | 1 (Ref.) |
|  | Yes | 16905 | 716 | 6.55864 | 1.23 (1.14-1.33) | 19225 | 793 | 6.43543 | 1.18 (1.10-1.27) | 33474 | 1356 | 6.15276 | 1.21 (1.14-1.28) |
| **Regular exercise** |  |  |  |  | P_int_ = 0.77 |  |  |  | P_int_ = 0.36 |  |  |  | P_int_ = 0.66 |
| **No** | No | 1741891 | 52065 | 4.39399 | 1 (Ref.) | 1728708 | 51043 | 4.33584 | 1 (Ref.) | 1541899 | 43547 | 4.14727 | 1 (Ref.) |
|  | Yes | 253002 | 11246 | 6.83832 | 1.17 (1.14-1.19) | 266185 | 12268 | 7.12694 | 1.18 (1.16-1.20) | 452994 | 19764 | 6.60221 | 1.20 (1.18-1.23) |
| **Yes** | No | 457026 | 13781 | 4.35149 | 1 (Ref.) | 452381 | 13532 | 4.31331 | 1 (Ref.) | 406005 | 11716 | 4.16157 | 1 (Ref.) |
|  | Yes | 60771 | 2433 | 5.94572 | 1.16 (1.11-1.21) | 65416 | 2682 | 6.11072 | 1.15 (1.11-1.20) | 111792 | 4498 | 5.91158 | 1.22 (1.17-1.26) |
| **Income** |  |  |  |  | P_int_ = 0.56 |  |  |  | P_int_ = 0.05 |  |  |  | P_int_ = 0.001 |
| **Top 80%** | No | 1739310 | 51963 | 4.36553 | 1 (Ref.) | 1727381 | 51108 | 4.31893 | 1 (Ref.) | 1541796 | 43595 | 4.12768 | 1 (Ref.) |
|  | Yes | 244755 | 10901 | 6.75647 | 1.16 (1.14-1.19) | 256684 | 11756 | 6.98539 | 1.16 (1.14-1.19) | 442269 | 19269 | 6.52122 | 1.20 (1.18-1.23) |
| **bottom 20%** | No | 459607 | 13883 | 4.45955 | 1 (Ref.) | 453708 | 13467 | 4.37791 | 1 (Ref.) | 406108 | 11668 | 4.23701 | 1 (Ref.) |
|  | Yes | 69018 | 2778 | 6.30876 | 1.18 (1.13-1.23) | 74917 | 3194 | 6.69162 | 1.21 (1.17-1.26) | 122517 | 4993 | 6.2443 | 1.22 (1.18-1.26) |

P_int_ = p for interaction

*Incidence rates were calculated per 1,000 person-years.

Adjusted for age, sex, BMI, low-income level, smoking status, alcohol consumption status, regular physical activity, hypertension, dyslipidemia, chronic kidney disease, heart failure, obstructive sleep apnea, thyroid disease, fasting glucose, DM duration, insulin use, and oral hypoglycemic agents.

Abbreviations; AF, atrial fibrillation; IR, Incidence rate; CI, confidence interval; HR, hazard ratio.

**II. Additional file Figure Legends**

Supplementary Figure 1. After sensitivity analysis, hazard ratios with 95% confidence intervals and incidence rate of new-onset AF for each mental disorder.

Abbreviation: AF, atrial fibrillation; HR, hazard ratio; CI, confidence interval.
