# Supplementary figures and images for: Impact of mental disorders on the risk of atrial fibrillation in patients with diabetes mellitus: a nationwide population-based study
Source: Cardiovasc Diabetol. 2022 Nov 17;21:251. doi: 10.1186/s12933-022-01682-7 (PMC9673441; doi:10.1186/s12933-022-01682-7)

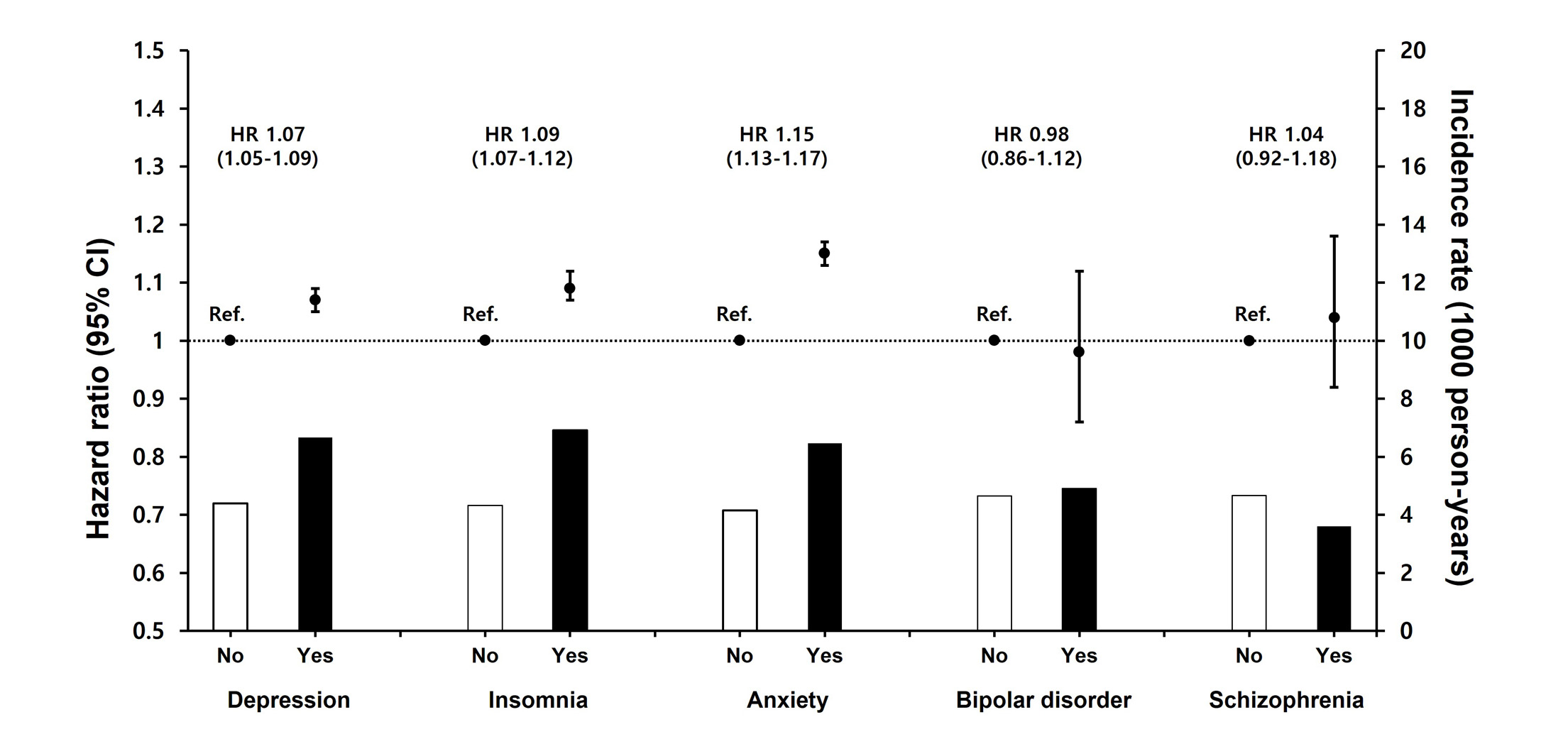

Supplement: Supplementary file 2 — Additional file 2: Figure S1. After sensitivity analysis, hazard ratios with 95% confidence intervals and incidence rate of new-onset AF for each mental disorder. [file 12933_2022_1682_MOESM2_ESM.jpg]
